# Effect of *Cymbopogan citratus* Fibre on Physical and Impact Properties of Thermoplastic Cassava Starch/Palm Wax Composites

**DOI:** 10.3390/polym15102364

**Published:** 2023-05-18

**Authors:** Zatil Hafila Kamaruddin, Ridhwan Jumaidin, Zatil Hazrati Kamaruddin, Muhammad Rizal Muhammad Asyraf, Muhammad Rizal Razman, Tabrej Khan

**Affiliations:** 1 Fakulti Kejuruteraan Mekanikal, Universiti Teknikal Malaysia Melaka, Hang Tuah Jaya, Durian Tunggal 76100, Melaka, Malaysia; 2 German-Malaysian Institute, Jalan Ilmiah Taman Universiti, Kajang 43000, Selangor, Malaysia; 3 Fakulti Teknologi Kejuruteraan Mekanikal dan Pembuatan, Universiti Teknikal Malaysia Melaka, Hang Tuah Jaya, Durian Tunggal 76100, Melaka, Malaysia; 4 Engineering Design Research Group (EDRG), Faculty of Mechanical Engineering, Universiti Teknologi Malaysia, Johor Bahru 81310, Johor, Malaysia; 5 Centre for Advanced Composite Materials (CACM), Universiti Teknologi Malaysia, Johor Bahru 81310, Johor, Malaysia; 6 Research Centre for Sustainability Science and Governance (SGK), Institute for Environment and Development (LESTARI), Universiti Kebangsaan Malaysia, UKM Bangi 43600, Selangor, Malaysia; 7 Department of Engineering Management, College of Engineering, Prince Sultan University, Riyadh 11586, Saudi Arabia

**Keywords:** starch, *Cymbopogan citratus* fibre (CCF), thermoplastic cassava starch

## Abstract

*Cymbopogan citratus* fibre (CCF) is an agricultural waste plant derived from a natural cellulosic source of fibre that can be used in various bio-material applications. This paper beneficially prepared thermoplastic cassava starch/palm wax blends incorporated with *Cymbopogan citratus* fibre (TCPS/PW/CCF) bio-composites at different CCF concentrations of 0, 10, 20, 30, 40, 50 and 60 wt%. In contrast, palm wax loading remained constant at 5 wt% concentration using the hot moulding compression method. TCPS/PW/CCF bio-composites were characterised in the present paper via their physical and impact properties. The addition of CCF significantly improved impact strength by 50.65% until 50 wt% CCF loading. Furthermore, it was observed that the inclusion of CCF resulted in a little decrement in biocomposite solubility compared to neat TPCS/PW biocomposite from 28.68% to 16.76%. Water absorption showed higher water resistance in the composites incorporating 60 wt.% fibre loading. The TPCS/PW/CCF biocomposites with different fibre contents had 11.04–5.65% moisture content, which was lower than the control biocomposite. The thickness of all samples decreased gradually with increasing fibre content. Overall, these findings provide evidence that CCF waste can be utilised as a high-quality filler in biocomposites due to its diverse characteristics, including improving the properties of biocomposites and strengthening their structural integrity.

## 1. Introduction

Over the past two decades, demand for agriculturally based products has grown due to environmental concerns and the awareness that petroleum resources are limited [1,2]. Polymers derived from biological sources have often been raised as the most promising solutions since they are renewable and biodegradable [3,4,5,6]. The development of organic, renewable, and biodegradable materials has been driven by the escalating amount of plastic trash that threatens the environment [7]. Sustainable development and the absence of damaging carbon emissions during processing and after destruction are desirable properties of prospective materials [8,9,10]. Excess plastic garbage is too much for recycling programmes because it is difficult to distinguish between polymers and eco-friendly plastics [11,12]. It is urgent to provide eco-friendly packaging items at comparable prices to preserve our planet, since synthetic plastic packaging pollution has exacerbated the issue of environmental destruction. In order to solve this recurring issue brought on by non-biodegradable plastics, natural biopolymers such as starch-based composites are being investigated as potential alternatives to conventional plastics [13,14,15].

Similar traits to conventional synthetic fibre-reinforced composites can be seen in natural fibre-reinforced composites. Due to considerable environmental concerns, they have been considered an alternative to organic and inorganic fillers and fibres [16,17]. These natural-source polymers can be found in various plant parts, such as grass [18], stalks [19], and leaves [20]. Hemicellulose, cellulose, and lignin are all examples of natural fibres [21]. Cellulose and hemicellulose components are all hydrophilic, whereas lignin is slightly hydrophobic [22,23]. Natural fibres are desirable filler materials for polymer composites because of their low manufacturing costs, particular acceptable characteristics, energy consumption, and environmentally friendly nature [24,25,26]. Natural fibres were incorporated to reinforce starch-based polymers, significantly impacting the materials’ physical and impact qualities. These were primarily attributable to cellulose and starch’s close structural resemblance and connection [27,28].

Starch, a form of carbohydrate, is one of the natural polymers that has received attention for use in the production of biodegradable polymers. Starch is generally composed of linear and branched chains of glucose molecules known as amylose and amylopectin, respectively [29]. Starch is combined with a plasticiser and fibre reinforcement and then subjected to high-temperature compression moulding to produce thermoplastic starch. Native starch can be transformed into a thermoplastic material by adding plasticising agents such as sorbitol, xylitol and glycerol. Incorporating plasticisers results in reduced intermolecular forces, increased mobility of polymer chains, and decreased glass transition temperature [30]. In our prior studies, we discovered that combining thermoplastic starch and palm wax had better properties than biopolymer blends [31,32]. As well, palm wax is considered an excellent matrix because it may reduce starch’s hydrophobicity, improving processability.

The use of starch in both food and non-food products has been widespread. Since starch-based composites have poor mechanical efficiency, particularly impact strength, improving their qualities is a big challenge [33,34]. Numerous blending and compositing techniques have been devised to enhance these mechanical properties, such as mixing with other polymers or strengthening natural fillers [35,36,37]. Various additional agents support the interaction between starch and other components. In order to build polymer blend composites and biocomposites, filler reinforcement added to starch-based polymers has recently received more attention [38,39]. It was established that using natural fillers to reinforce materials with specific capabilities and add new features is a successful strategy. Although pure thermoplastic starch has many benefits, it also has some drawbacks that restrict the range of its potential uses. These drawbacks include limited water barriers, mechanical strength, and long-term stability [40,41,42]. Thus, modifications are typically needed to make thermoplastic starch useful in actual applications. The utilization of natural fibers as reinforcement for thermoplastic starch is an intriguing approach that could be utilized to solve these shortcomings. The mechanical properties of natural fibers, when combined with thermoplastic starch, are obviously improved due to the chemical similarity of starch and plant fibers [43]. In addition, thermoplastic starch can be used in combination with other natural polymers to reduce the downsides of this biopolymer while maintaining the material’s biodegradability [15]. Results showed that adding palm wax significantly improved the mechanical characteristics of a cassava starch/palm wax thermoplastic blend [31] and improved the biopolymer’s performance. Additionally, it contributed to the biopolymer’s distinctiveness as an edible food coating and food packaging material.

Lemongrass, also called *Cymbopogan citratus,* is one of the Poaceae family’s aromatic plants. These natural crops are grown in the tropical and semitropical areas of Africa, Asia, South America, and India [32]. In Malaysia, *Cymbopogon citratus* fibre (CCF) is becoming more well-liked as a natural resource that can help create ecologically friendly resources. The leaves of *Cymbopogan citratus* grow directly from the earth and have short rhizomes. All parts of the *Cymbopogan citratus* plant, including the leaves, are abundantly produced since the plant is simple to grow. The leaves have a glabrous surface and a greenish interior and can reach a length and width of about 50 and 1.5 cm, respectively [44]. The leaves of the *Cymbopogon citratus* plant are thrown away as trash in Malaysia, where the stalks are most frequently utilised as a constituent to flavour cuisine [44]. Due to the significant volume of garbage created, various trash reduction attempts have been conducted by preventing disposal and reusing waste. CCF has recently been employed as an adsorbent from aqueous solutions to remove methylene blue dye, as a raw material for making paper and pulp, and broadly in medicinal activities [45,46]. In addition, CCF is one of the substitute materials used to manufacture polymer composites as a reinforcing agent. The fibre of *Cymbopogan citratus* contains a significant quantity of cellulose, making it a source of cellulose fibre, according to research [47].

Although research has been reported on using Cymbopogan species from China in synthetic polymers, none was discovered on the characterisation of Cymbopogan citratus fibre from Malaysia in a thermoplastic cassava starch/palm wax matrix. As a result, the primary objective of this research topic is to investigate the effects of Malaysian-sourced Cymbopogan citratus fibre on the impact resistance and physical properties of thermoplastic cassava starch/palm wax composites. This study aims to produce decomposable materials that reduce pollution and are more eco-friendly.

## 2. Materials and Methodology

### 2.1. Materials

CCF, with contents of cellulose (37.56%), hemicellulose (29.29%), lignin (11.14%), and ash (4.28%), was collected from a rural area located in Beranang, Selangor (West Malaysia). The extraction of CCF was performed according to our previous research [48] via the water-retting process. Antik Sempurna Sdn. Bhd., based in Selangor, Malaysia, provided the food-grade cassava starch used in this investigation. The palm wax in the commercial grade was provided by Green & Natural Industries Sdn. Bhd and the glycerol (99.5% purity) was purchased from QRec Chemicals Sdn, Selangor, Malaysia.

### 2.2. Sample Preparation of Biocomposites

The thermoplastic cassava starch (TPCS) preparation was made by combining 5% palm wax with 65% starch and 30% glycerol. The mixture was then blended at room temperature for 5 min at 1200 rpm using a Panasonic Dry Mixer MX-GM1011. The materials were subjected to thermo-pressing at 150 °C for 30 min using a Malaysian-made Technopress-40HC-B Plastic Hydraulic Moulding Press under a weight of 10 tonnes to produce plates with a thickness of about 3 mm. Similar techniques were applied to create TPCS/PW/CCF composites. The matrix’s characteristics were altered by introducing various CCF ratios ranging from 0 to 60 wt.%. The specimens were immediately put in a desiccator with silica gel to avoid unforeseen water absorption before conditioning.

### 2.3. Density

The ASTM D1895 [49] standard was followed to determine the biocomposite material density of the samples (10 mm × 10 mm × 3 mm), which were fabricated and dried for 24 h at 105 °C in the oven. The specimens were then positioned in a desiccator with silica gel in granulated form, and their weights and volumes were calculated using an electronic densimeter and weighing balance. Equation (1) was used to determine the density value.

(1) $$\mathrm{Density}\;(g/{\mathrm{cm}}^{3})=\frac{\mathrm{Mass}\;\left(g\right)}{\mathrm{Volume}\;\left({\mathrm{cm}}^{3}\right)}$$

### 2.4. Moisture Content of Composite Materials

Moisture content analysis was performed for five samples that were prepared and heated in the oven for 24 h at 105 °C. The initial weight (W_i_, grammes) and the final weight (W_f_, grammes) after heating are needed to calculate the moisture content [50]. Equation (2) was utilised to compute the moisture content.

(2) $$\mathrm{Moisture}\;\mathrm{content}\;(\%)=\frac{W_{i}-W_{f}}{W_{i}}\;\times \;100\;$$

### 2.5. Water Absorption

Five samples (10 mm × 10 mm × 3 mm) were dried for 24 h at 105 ± 2 °C in an air-circulating oven to remove existing moisture. The test samples were fully submerged in water for 2 h at ambient temperature (23 ± 1 °C). The initial weight (W_i_) and final immersion weight (W_f_) were calculated for determining the water absorption rate using Equation (3).

(3) $$\mathrm{Water}\;\mathrm{absorption}\;(\%)=\frac{W_{i}-W_{f}\;}{W_{i}}\;\times \;100$$

### 2.6. Thickness Swelling

Analysis of the samples’ swelling was conducted based on the amended procedure by Jawaid et al. [51]. Five samples with measurements of 10 mm × 10 mm × 3 mm were prepared for thickness swelling calculation. Specimens were then dried in a dry laboratory oven at 105 °C for 24 h. Each sample was measured for thickness before testing (T_i_). After that, each sample was submerged in 30 mL of distilled water for 2 h at room temperature (23 ± 1 °C). During the immersion process, the starting and final thicknesses, T_i_ and T_f_, were measured using a Mitutoyo brand digital vernier calliper with a precision of 0.01 cm. This allowed for more accurate reading. Equation (4) summarizes the steps used to calculate the samples’ thickness swelling ratio:

(4) $$\mathrm{Thickness}\;\mathrm{swelling}\;(\%)=\frac{T_{i}-T_{f}}{T_{i}}\;\times \;100$$

### 2.7. Impact Testing of Composites Material

Analysis via Izod impact tests was performed using ASTM D256 [52] with 50 ± 5% relative humidity and temperature of 23 ± 1 °C, and five replicates of each sample with dimensions of 13 mm (W) × 3 mm (T) × 60 mm (L) were set up. Victor Equipment Resources Sdn. Bhd. (Subang Jaya, Malaysia) provided testing using a digital pendulum impact tester. Before testing, all samples were preconditioned for two days and then processed at 53% RH. The results of the impact properties were obtained by taking the average of the data using Equation (5).

Impact strength = Impact energy (J)/area (mm^2^) (5)

### 2.8. Water Solubility of Composite Materials

The procedure described by Zhang et al. [53] was used to measure the samples’ water solubility with a few minor adjustments. Five samples (10 mm × 10 mm × 3 mm) were cut and dried in a dry laboratory oven for 24 h at 105 °C 2 and the initial dry matter of each sample was noted as W_i_. Each sample was immersed in 30 mL of distilled water while stirring vigorously. The undissolved portions of the sample were then taken out of the laboratory cup after a 24-h immersion, and any remaining water on the sample’s surface was wiped away using filter paper. The samples were then once again dried for 24 h at 105 °C to produce the final dried sample, indicated as W_f_. Equation (6) was used to calculate the samples’ water solubility.

(6) $$\mathrm{Water}\;\mathrm{solubility}\;(\%)=\frac{W_{i}-W_{f}\;}{W_{i}}\;\times \;100$$

### 2.9. Statistical Analysis of Composites Material

Analysis of variance (ANOVA) was used for statistical analysis of the experimental results in SPSS. Duncan’s test was used to compare the means with a significance level of 0.05 (*p* < 0.05).

## 3. Results and Discussions

### 3.1. Density

**Figure 1:**
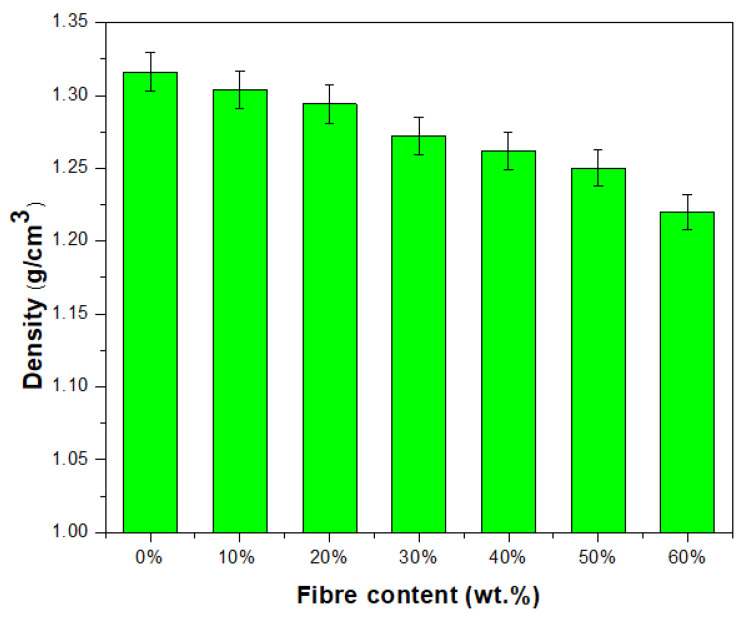
Density of TPCS/PW/CCF biocomposites.

Biocomposites of TPCS/PW/CCF were produced by varying the CCF loading from 0% to 60% of fibre. There was a slightly significant difference in TPCS/PW/CCF biocomposite density. However, when fibre was introduced, it was discovered that the density value of biocomposite reduced as the fibre loading increased, as illustrated in Figure 1, ranging from 1.31 ± 0.01 to 1.22 ± 0.01 g/cm^3^. This finding might be related to the low density of the added CCF, which is known to be 0.25 ± 0.002 g/cm^3^, relative to the TPCS/PW control sample (CCF0), which had a density value of 1.31 ± 0.01 g/cm^3^. This affected the overall density of the bio-composite that was produced. Considering the density value of TPCS/PW/CCF, it can be noticed that the density of samples decreased by 6.87%. This specifies that the decreasing trend in density value might be associated with the reduction in the proportion of biocomposite mass when CCF is added, as the percentage of the TPCS/PW matrix is reduced while the volume remains constant [28]. As a result, reduction in biocomposite mass had a corresponding effect on the density values of the samples. This finding agreed with a previous study on developing Dioscorea hispida fibre-reinforced Dioscorea hispida starch, where the biocomposites’ density values decreased as fibre loading was added [29]. Meanwhile, a similar result was reported when developing wood apple shell-reinforced epoxy composites. The density of the composites decreasing as the amount of filler used increased might be due to the lighter density of the filler material [54]. In terms of practicality, the decrease in bio-composite density when fibre loading is increased makes a substantial contribution by providing lightweight materials [55].

### 3.2. Moisture Content

**Figure 2:**
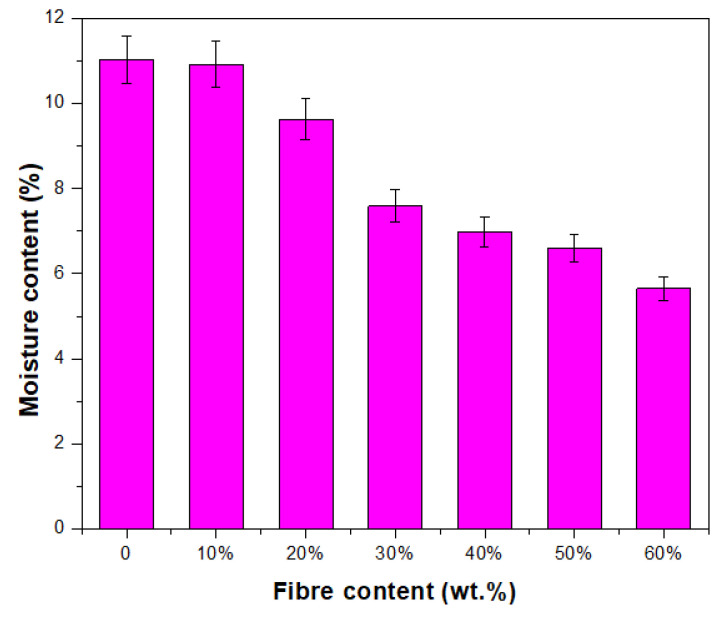
Moisture content of TPCS/PW/CCF biocomposites.

Thermoplastic starch exhibits a high moisture content, which is caused by the inherent hydrophilic nature of native cassava starch, and results in poor moisture barrier capabilities. In spite of this, thermoplastic starch’s resistance to water can be increased by the inclusion of components that are either hydrophilic or hydrophobic. This study investigated the effect of Cymbopogan citratus fibre on thermoplastic cassava starch/palm wax composites’ moisture content. The moisture content of the thermoplastic starch/palm wax composite without *Cymbopogan citratus* fibre was the highest, with absorption rates of 11.04%, which might be due to the strong hydrogen interactions between the hydroxyl groups in starch and the free water molecules [56]. The TPCS/PW/CCF biocomposites with different fibre contents had moisture contents of 11.04–5.65%, which was lower than that of the control biocomposite sample (11.04%), as illustrated in Figure 2. However, the increase in CCF content from 10 to 60 wt.% resulted in a slight reduction in the moisture content of the TPCS/PW/CCF samples. This could be explained by the strong hydrogen bonds between the Cymbopogan citratus fibre and the starch matrix, reducing the number of accessible hydroxyl groups.

Consequently, the biocomposites have increased resistance to moisture. This result agrees with the previous study’s finding on the effect on moisture content of cassava starch foam with the addition of natural fibre and chitosan [57]. Likewise, Soykeabkaew et al. [58] reported that similar results occurwhen jute and flax are added to tapioca starch foam, decreasing the amount of moisture content. This could be related to the hydrophobic nature of CCF, especially when compared to the CCF 0% sample. Although all-natural fibre is classified as hydrophilic, it is not as hygroscopic as TPCS. This might be due to the presence of lignin and wax in CCF composition, which makes it comparatively hydrophobic and gives it better water-resistant properties than TPCS [59]. In contrast, palm wax is considered a superior matrix since it can reduce starch hydrophobicity, which improves processability [31,60].

### 3.3. Water Absorption of Composites

**Figure 3:**
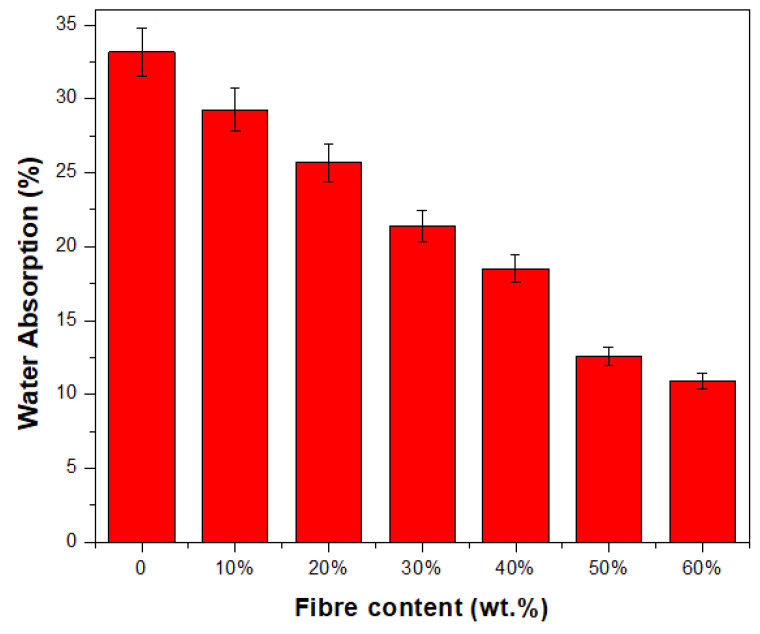
TPCS/PW/CCF biocomposites’ water absorption.

Water absorption is crucial for many applications of TPS products. The results of the water absorption tests performed on TPCS/PPW/CCF biocomposites are shown in Figure 3. In general, water absorption followed the same trend as moisture content, which exhibited decreasing values when fibre content is increased. In this study, the neat TPCS/PW biocomposite showed the highest water absorption at 33.15%. However, the absorption rate appeared to be reduced with CCF reinforcement, which reached 10.90% with 60 wt.% loading.

The ability of fibres to produce strong interfacial bonding, which prevents water from penetrating through the matrix, may play a role in the decrease in absorption rate in the presence of CCF [61]. In addition, the presence of lignin as the main component, in conjunction with wax and fatty substances, in fibre enhances the ability of composites to withstand the effects of water [61,62]. A prior study also reported that the gradual loading of sugar palm fibre into TPS-based hybrid composites resulted in a considerable reduction in the water absorption rate [63]. Meanwhile, a previous study by Sarifuddin et al. [64] on developing thermoplastic sago starch reinforced kenaf core fibre biocomposites reported low water absorption when adding kenaf core fibre into the samples. This was associated with the fibre having a less hydrophilic nature than the starch. The higher the fibre loading, the lower the amount of absorbed water. The strong hydrogen bonding between the matrix phase and the fibre phase can be attributed to the overall lower water absorption of the material. However, it is clear that the CCF-reinforced starch biocomposite is less water-resistant than the control CCF samples.

### 3.4. Thickness Swelling

**Figure 4:**
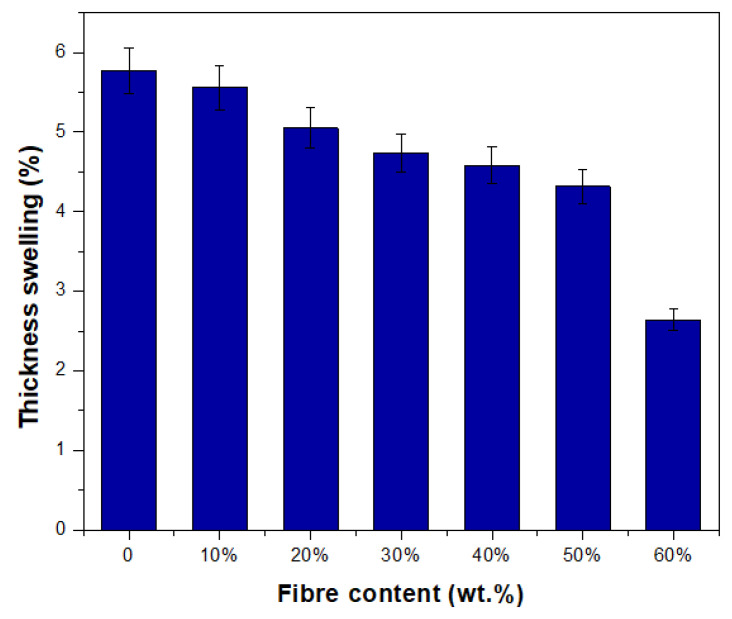
Thickness swelling of TPCS/PW/CCF biocomposites.

A material’s dimensional stability can be evaluated by determining the degree to which it swells or contracts as a direct result of the movement of moisture in the material. The thickness swelling ratio of TPCS/PW/CCF composites was evaluated to determine changes in TPCS/PW dimensional stability following the introduction of CCF. Figure 4 illustrates the thickness swelling percentage for TPCS/PW with the inclusion of CCF after immersion for 2 h. The thickness of all samples decreased gradually with increasing fibre content. In comparison to the TPCS/PW control sample, the sample containing 60% CCF content exhibited the lowest water uptake and thickness swelling after 2 h of immersion, decreasing by 54.24%. This finding can be attributed to the presence of fibre in the composites, which possesses a more rigid structure than starch, providing higher dimensional stability to the composites [65]. This study’s findings agree with those of a prior investigation into composites made of cassava starch and green coir fibres [66]. Additionally, similar findings were reported when developing a cassava/sugar palm fibre-reinforced cassava starch hybrid. In this study, the incorporation of sugar palm fibre made the sample swell less and influenced the interaction between the polymer chains, which could reduce the polymer’s ability to swell. In each sample, increasing the fibre concentration resulted in a decrease in swelling behaviour. The sample that contained a low fibre loading was more swollen than the ones that contained a high fibre loading [67].

### 3.5. Impact Testing of Composites

**Figure 5:**
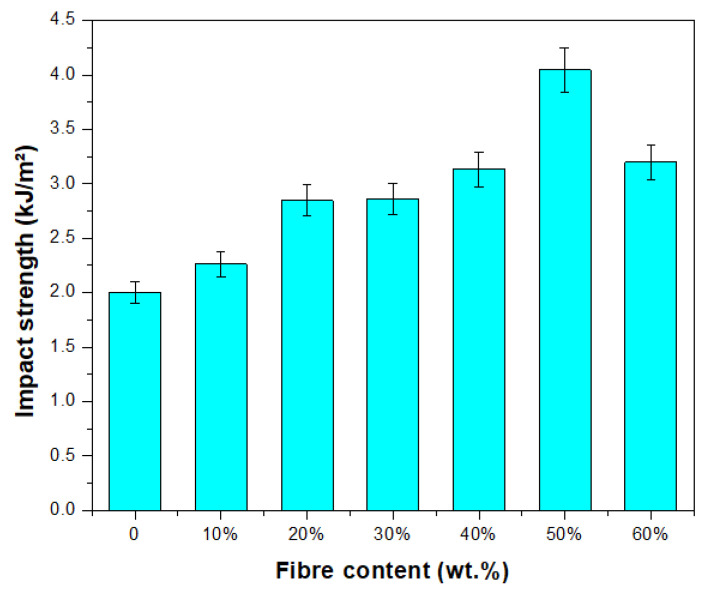
Impact strength of TPCS/PW/CCF biocomposites.

The impact strength of the biocomposites was also determined to measure the material’s impact resistance, indicating the capability to endure an unexpectedly applied load. Figure 5 demonstrates the TPCS/PW/CCF’s impact strength with different fibre contents. Analysis of variance (ANOVA) of the impact characteristics is presented in Table 1. This indicates that there is a statistically significant difference (*p* < 0.05) between the mean impact strength from one level of composites to another. In general, the findings of the impact test results demonstrate the highest impact strength at 50 wt.% fibre loading. Significant improvement (*p* < 0.05) was evidenced in the impact properties of the composite when incorporated with *Cymbopogan citratus* fibre. Failure of a fibre composite to withstand impact loading can be attributed to several other factors, including fibre pull-out, matrix fracture, fibre breakage, and debonding of the fibres from the matrix [68]. It could be observed that the TPCS/PW/CCF biocomposite had significantly increased impact strength by 50.65% (*p* < 0.05) with increasing fibre content from (0 to 50 wt.%). This finding might be associated with the strong interfacial bonding at fibre–matrix interfaces, which improved energy absorption during impact loading [69]. However, impact resistance declined after increasing reinforcement from 50 to 60 wt.%. The reduction in impact strength at higher fibre contents might be attributed to the higher rigidity of the materials, which led to more brittle properties and, as a result, a decreased capacity to absorb the impact energy [31]. In our prior studies, we discovered that the results revealed that elongation at break values increased from 40 to 50 wt.% as a result of the addition of *Cymbopogan citratus* fibre loading; however, an increase in the amount of *Cymbopogan citratus* fibre up to 60 wt.% caused a decrease in elongation at break values. Overall, the inclusion of *Cymbopogan citratus* fibre content resulted in a decrease in the molecular mobility of the TPCS matrix, which produced biocomposite materials with a greater degree of rigidity [48]. A similar result was observed in a study on the development of date palm-reinforced epoxy composites. A significant improvement in impact strength was found at 50% date palm fibre reinforced epoxy composites in comparison to loadings of 40% and 60%, which can be primarily attributed to better adhesion of the date palm fibre with the epoxy matrix in order to overcome the high impact stress/load. Furthermore, the increase in impact strength might be associated with enhanced stress capability, which would limit the contribution of fibre-related mechanisms such as fibre pull out [70].

### 3.6. Water Solubility of Composites

**Figure 6:**
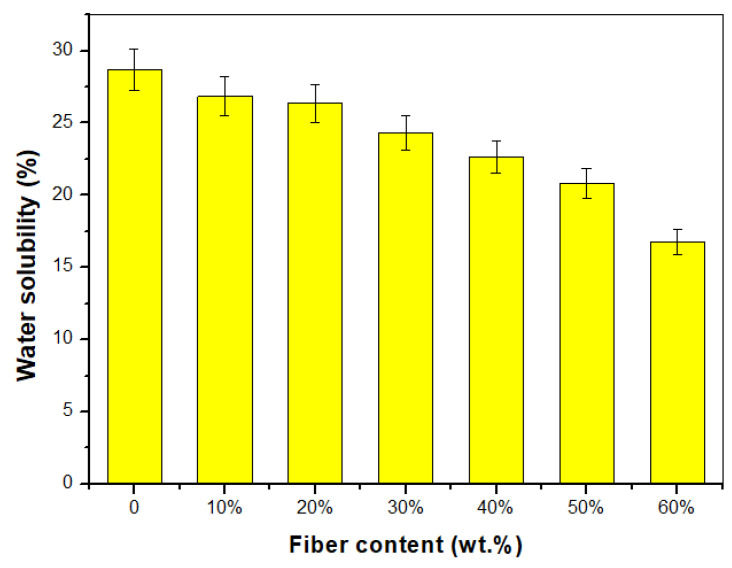
Water solubility of TPCS/PW/CCF biocomposites.

The solubility of samples in water is one of the essential properties worthy of consideration in many applications. Applications that require moisture and water loss protection must have low water solubility. Figure 6 shows the TPCS/PW/CCF biocomposites’ water solubility. This solubility demonstrates the effect of water immersion with continuous stirring on the composite samples. The inclusion of CCF resulted in a little decrement in biocomposite solubility compared to neat TPCS/PW biocomposite, from 28.68% to 16.76%. This phenomenon could be attributed to the role of the fibre, which produces a network that tightly keeps composites together and prevents the dissolution of composites by lowering the solubility of the samples [55]. Additionally, the solubility of samples reduced with increasing fibre content, which might be due to fibre’s capacity to resist water diffusion and enhance composite integrity by limiting water penetration, thus reducing sample solubility [63]. This observation agrees with previous findings on the development of a cornhusk/sugar palm fibre reinforced corn starch-based hybrid, which reported that adding sugar palm fibre decreased the solubility of the samples [61]. The result supports this study’s water absorption, moisture content, and thickness swelling findings.

## 4. Conclusions

Utilising hot pressing, a novel biocomposite made of thermoplastic cassava starch and varying concentrations of *Cymbopogan citratus* fibre (CCF) was created. Its water barrier behaviour and impact qualities were studied. The experimental results revealed that the inclusion of CCF resulted in a little decrement in biocomposite solubility compared to neat TPCS/PW biocomposite, from 28.68% to 16.76%, and a reduction in the absorption rate, which reached 10.90% with 60 wt.% loading. TPCS/PW/CCF biocomposites with different fibre contents had moisture contents of 11.04–5.65%, which was lower than the control biocomposite sample, and a slightly significant difference in biocomposite density. TPCS-reinforced CCF composites show promise as an alternative to non-environmentally friendly polymers and composites, with better water barrier and impact performance.

## Figures and Tables

**Table 1:** Analysis of variance (ANOVA) summary of TPCS/PW/CCF composites.

Variables	df	Impact Strength
Mixture	6	0.00 *

Note: * Significantly different at *p* < 0.05.

## Data Availability

The data is available within the article.
